# Does anxiety cause angina recurrence after percutaneous coronary intervention in patients with STEMI?

**DOI:** 10.3389/fcvm.2023.1283182

**Published:** 2023-10-31

**Authors:** Zhipeng Zhang, Xing Wei, Jing Wei, Yuhui Wang, Chunmiao Luo

**Affiliations:** ^1^ Department of Cardiology, The Second People’s Hospital of Hefei, Hefei Hospital Affiliated to Anhui Medical University, Hefei, Anhui, China; ^2^ The Fifth Clinical School of Medicine, Anhui Medical University, Hefei, China

**Keywords:** anxiety, STEMI, percutaneous coronary intervention, angina, follow-up

## Abstract

**Background:**

Statistics show that approximately 70% of patients with acute ST-segment elevation myocardial infarction (STEMI) experience relief from chest pain symptoms within 48 h post-percutaneous coronary intervention (PCI). However, over 30% of these patients still suffer from angina post-PCI during their hospital stay and after discharge. Although the interrelation between cardiovascular diseases and psychological states, notably anxiety and stress, has been extensively studied and acknowledged, the specific influence of anxiety disorders on post-PCI clinical outcomes for STEMI patients, especially the recurrence of angina, remains undefined.

**Methods:**

This study included a total of 324 STEMI patients who underwent PCI treatment due to chest pain in our hospital. Baseline and surgical data for all patients were collected. During their hospital stay, patients’ emotional states were assessed using the Hamilton Anxiety Scale, while angina was evaluated using the Seattle Angina Questionnaire. All patients were followed up for 6 months post-discharge to gather clinical data and outcomes, analyzing whether anxiety disorders would affect the recurrence of angina post-PCI in STEMI patients.

**Results:**

Out of the 324 patients, 82 experienced recurrent angina symptoms within 6 months post-PCI discharge. Compared to the non-recurrence group, the recurrence group showed statistically significant differences in anxiety levels. Other differing factors included the spouse's health status, cardiac Killip classification, severity of coronary lesions, and the state of the coronary microcirculation. After utilizing propensity score matching to eliminate inherent biases between the two groups at a 1:1 ratio, the COX regression analysis indicated that a patient's anxiety status is a risk factor for the occurrence of angina post-PCI in STEMI patients (HR = 2.094, 95% CI = 1.248–3.514, *P* = 0.005).

**Conclusion:**

Anxiety is a significant factor for short-term recurrence of angina post-PCI in STEMI patients. This further confirms the crucial impact of mental health on cardiovascular wellness.

## 1. Introduction

Angina is a clinical syndrome resulting from an imbalance in the myocardial oxygen supply, leading to myocardial ischemia. The primary triggers for its onset are either an increased oxygen demand by the heart or a reduction in the blood flow through the coronary arteries. Clinically, reduced blood flow due to coronary lesions often precipitates angina, with the most severe type being Acute Myocardial Infarction (AMI) (1). Emotional distress, excessive fatigue, and poor lifestyle choices all play significant roles in triggering angina (2). Percutaneous Coronary Intervention (PCI) can quickly restore blood flow in obstructed vessels, effectively alleviating chest pain, reducing AMI mortality, and enhancing patient quality of life. Its efficacy in reducing myocardial ischemia is significantly superior to pharmacotherapy (3). However, not all patients experience complete relief from chest pain post-PCI; some face a recurrence shortly after discharge. Studies indicate (4) that the recurrence of anginal symptoms post-PCI is a risk factor for adverse cardiovascular events. Compared to those without recurrent pain, patients experiencing chest pain recurrence within a year post-PCI face medical costs that are 1.8 times higher (5). This highlights the need to further investigate and understand the myriad factors that may precipitate angina recurrence, to mitigate its impact on patient health and the healthcare system. Anxiety can directly impact cardiac function and potentially increase the risk of angina recurrence indirectly through influencing patient behavior and lifestyle choices. Hence, providing a more accurate risk assessment and management strategy, further optimizing the treatment and recovery of STEMI patients, and exploring the link between anxiety and angina recurrence becomes crucial.

## 2. Literature review

Through the exploration and review of existing literature, we find that anxiety has a dual impact on human health: on one hand, it can trigger various self-protective mechanisms in the body (such as preventing the recurrence of traumatic memories and enhancing immune responses), while on the other hand, excessive anxiety can lead to a series of severe pathological conditions (such as cytokine storms within the body, post-traumatic stress disorder, and myocardial infarction) (6). A study of notable significance was conducted in 2011 by Swedish scholar Falkstedt D and colleagues (7), which encompassed a sample of 49,321 men. The subjects were assessed for anxiety prior to their conscription and were followed up for 37 years. It was concluded that anxiety disorders are closely related to the onset of cardiovascular diseases and acute myocardial infarctions. A domestic study reflecting the beneficial impact of treating anxiety disorders for better prognosis of coronary heart disease was identified (8). In this study, patients with coronary heart disease and anxiety disorders were divided into two groups. The first group received conventional coronary heart disease drug treatment, while the second group received anti-anxiety medication in addition to the conventional treatment. After a 12-week follow-up, the study results indicated that both drug and exercise therapies can delay the progression of coronary heart disease in patients with anxiety disorders. This implies that addressing anxiety disorders in tandem with the primary disease can be highly beneficial. Rutger W (9) found through patient-centered personalized networks and remote cardiac monitoring that anxiety disorders impact the morbidity of coronary artery disease patients. Improving anxiety disorders can reduce the recurrence rate and mortality of discharged patients with coronary heart disease. There are also domestic and international reports on the impact of anxiety disorders on hypertension, heart failure, and other cardiovascular diseases (10, 11).

The mechanism by which anxiety affects cardiovascular diseases is still not clear. Some scholars propose that anxiety disorders might influence the development of cardiovascular diseases through pathways. The most accepted mechanism is that anxiety leads to hyperactivation of the hypothalamic-pituitary-adrenal axis and the sympathetic nervous system, increased release of plasma catecholamines, and endothelial damage, eventually causing arteriosclerosis, coronary artery disease, and acute coronary events. This can also explain the relationship between anxiety and hypertension. Some scholars believe that the cardiovascular effects of psychological stressors such as anxiety and depression are consistent (12). Acute anxiety and cardiovascular stress lead to increased resting heart rate reactivity, decreased heart rate variability, impaired baroreflex function, and increased ventricular repolarization variability. In summary, the hyperactive sympathetic nervous system and the hypothalamic-pituitary-adrenal axis release nerve impulses that control cardiac activity changes, a process that may increase the risk of cardiovascular diseases, further increasing the risks of cardiac ischemia, arrhythmias, and sudden cardiac death.

Though current theoretical knowledge offers explanations for how anxiety impacts cardiovascular diseases, there's a lack of corresponding clinical research both domestically and internationally to confirm these theories. In our clinical work, designing a scientific research plan to demonstrate how anxiety promotes the progression of cardiovascular diseases, and studying the relationship between post-PCI STEMI patient angina recurrence and anxiety, is a preferred choice within a controllable scope. Due to sample size limitations and the presence of errors, conventional statistics might not produce relatively accurate results. Propensity Score Matching (PSM) is an innovative method to some extent to overcome endogeneity bias and is highly recommended in academic circles. Therefore, this study uses the PSM method to explain the baseline data of the research subjects and further conducts statistical analysis.

## 3. Materials and methods

### 3.1. Study subjects

**Figure 1:**
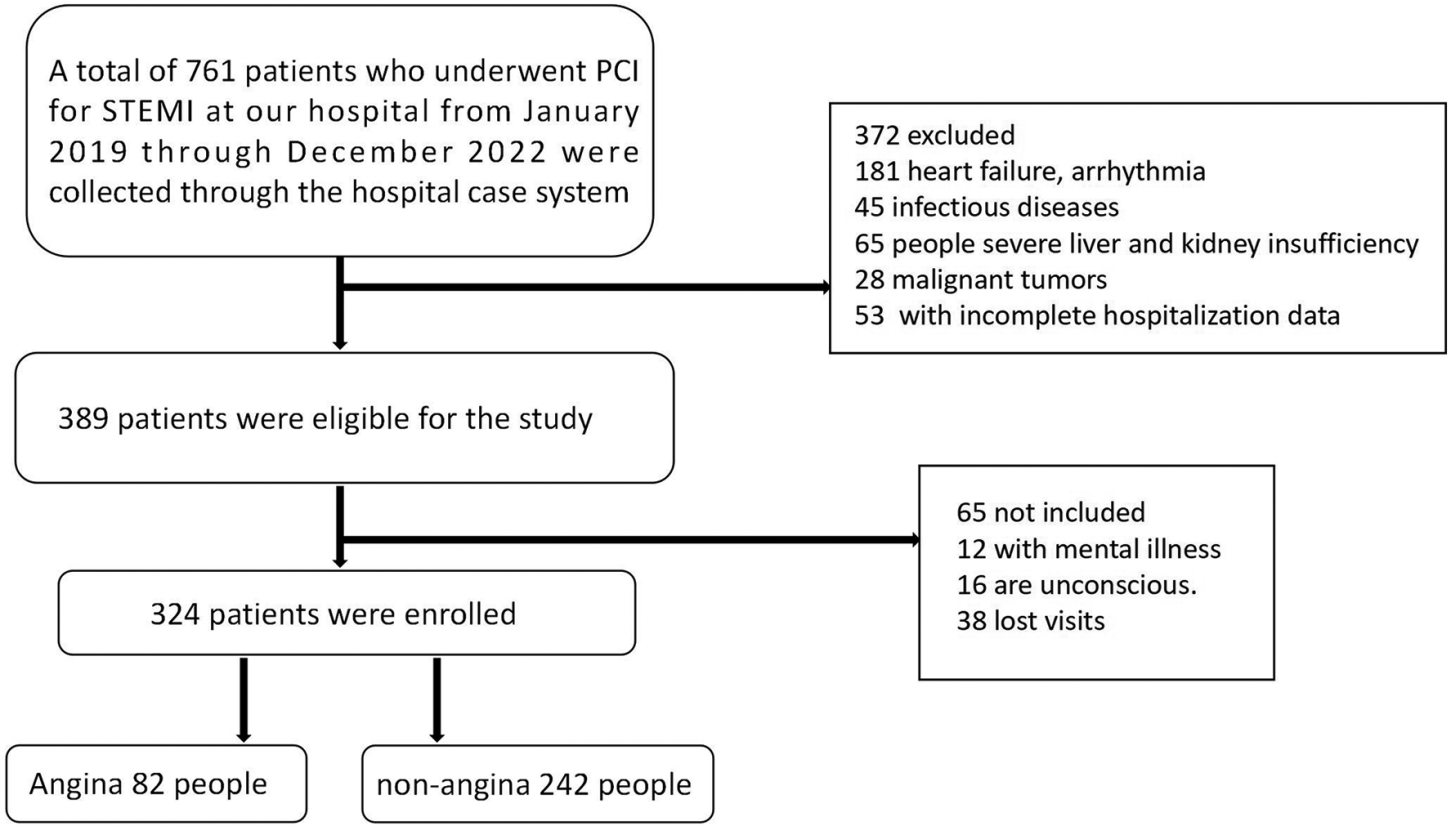
Study flow diagram.

This study included a total of 324 cases from January 2019 to December 2022, who visited our hospital due to chest pain, met the inclusion criteria, and had complete hospitalization records. All patients were admitted with evidence of STEMI based on a 12-lead bedside ECG and exhibited relief from chest pain symptoms upon discharge after undergoing PCI. A 6-month follow-up was conducted for all patients. Based on the follow-up results, patients were divided into two groups: chest pain recurrence group (Group I, *n* = 82) and non-recurrence group (Group II, *n* = 242). Inclusion criteria were: (1) Age ≥18 years; (2) Indications met for PCI; (3) Absence of angina symptoms at discharge post-PCI; (4) Clear consciousness. Exclusion criteria included: (1) Chronic heart failure, severe arrhythmias, or other cardiovascular diseases; (2) Infectious diseases; (3) Severe hepatic or renal dysfunction; (4) Malignant tumors; (5) Mental disorders; (6) Poor compliance or lost to follow-up (Figure 1).

### 3.2. Variable description

(1) Dependent variable: Recurrence of angina in patients. The Seattle Angina Questionnaire (SAQ) was used to score angina, with the scale consisting of 5 main sections and 19 items. After scoring each item, corresponding formulas were used to convert scores to standard points, ranging from 0 to 100. A higher score suggests milder clinical symptoms (13). In this study, an SAQ score between 75 and 100 was considered as no angina.
(2) Key independent variable: Patient's anxiety level. The Hamilton Anxiety Rating Scale (HAMA) was used to assess anxiety levels in both groups, with HAMA scores ranging from 0 to 56. A higher score indicates severe anxiety (14). In this study, a HAMA score >10 was defined as an anxiety disorder.
(3) Control variables: These primarily included patients’ baseline clinical data, surgical details, and postoperative medications. Data, such as gender, age, Body Mass Index (BMI), economic status, spouse's health condition, smoking history, hypertension, diabetes, and history of hyperlipidemia were retrospectively collected from the hospital's medical records system. Some continuous variables were transformed into categorical ones for statistical convenience. Information was gathered by the nursing department during patients’ hospital admission. Economic status was based on the average annual income of local residents, with those earning below 50% of the average considered poor and above 100% as wealthy. Spousal health status was differentiated based on their activity level, categorized as healthy, ill, or no spouse. Illnesses included stroke, Alzheimer's, arthritis, fractures, vision or mental disorders, heart disease, and chronic pulmonary diseases that limit activity. Before surgery, all patients were given a dual antiplatelet therapy combination of aspirin with either ticagrelor or clopidogrel. The time of preoperative chest pain was noted. During the operation, three experienced coronary intervention surgeons assessed the coronary lesions of the patients. Intraoperative data such as thrombolysis, stent implantation, and circulation status (evaluated by TIMI grading) were recorded. Unless contraindicated, patients were given dual antiplatelet therapy (aspirin with either ticagrelor or clopidogrel) and statins post-operation. Additionally, treatment options such as *β*-blockers, ACE inhibitors (ACEI)/Angiotensin II receptor blockers (ARB), nitrate vasodilators, etc., were administered as needed. Postoperative relief of chest pain symptoms within 36 h and in-hospital adverse events (excluding chest pain recurrence), such as severe hypotension, acute heart failure, arrhythmia, shock, and puncture site infection, were noted.

### 3.3. Follow-up details

Researchers followed up with all study participants for 6 months post-discharge through outpatient visits, patient chat groups, phone calls, and text messages. The average follow-up time was 150 days The main focus of the follow-up was the recurrence of angina within 6 months post-discharge and if the SAQ score met the angina criteria, noting the time of angina recurrence.

### 3.4. Statistical methods

Data were analyzed using SPSS 27.0 and R 4.3.1 software. The Shapiro-Wilk test was employed to check for the normality of continuous variables. Non-normally distributed data were expressed as median (P25, P75) and analyzed using the Mann-Whitney U test. Categorical variables were expressed in percentages and compared using the Chi-square test or Fisher's exact test. COX regression analysis was conducted to identify risk factors for post-PCI angina recurrence, calculating hazard ratios (hr) and 95% CI. The Kaplan-Meier method was used to plot survival curves, and the log-rank test compared differences between patient groups with varying clinical pathologies. The PSM method was employed to match certain baseline data and other significant differences, setting a caliper value of 0.02. Patients were matched in a 1:1 ratio based on closest scores. A *P*-value of <0.05 was considered statistically significant.

## 4. Results and analysis

### 4.1. Comparison of baseline data, surgical information, and postoperative medication between the two groups of patients. A total of 324 subjects were included in this study. Based on follow-up results, 82 individuals experienced recurrent angina within 6 months.

For baseline data, there were no significant differences between the two groups in terms of gender, age, BMI, economic background, and personal history, including smoking, hypertension, diabetes, hyperlipidemia, etc. (*P* > 0.05). Patients with recurrent angina had a higher prevalence of anxiety disorders and their spouses were in poorer health, primarily reflected in a higher likelihood of their spouses having illnesses. The difference was statistically significant (*P* < 0.05), as shown in Table 1.

**Table 1:** Comparison of baseline data between the two groups of patients.

Characteristics	Totality	Group I	Group II	*P*
Sex				0.442
Male	252 (77.8)	61 (74.4)	191 (78.9)	
Female	72 (22.2)	21 (25.6)	51 (21.1)	
Age				0.307
≥65	160 (49.4)	46 (56.1)	118 (48.8)	
<65	164 (50.6)	36 (43.9)	124 (51.2)	
BMI				0.701
>24	179 (55.2)	47 (57.3)	132 (54.2)	
≤24	145 (44.8)	35 (42.7)	110 (45.5)	
Economic situation				0.931
Normal	243 (75)	63 (76.8)	180 (74.4)	
Poor	42 (13)	10 (12.2)	32 (13.2)	
Rich	39 (12)	9 (11.0)	30 (12.4)	
Spouse				<0.001
Well	208 (64.2)	38 (46.3)	170 (70.2)	
Unwell	94 (29)	36 (43.9)	58 (24.0)	
None	22 (6.8)	8 (9.8)	14 (5.8)	
Smoking				0.610
Yes	167 (51.5)	40 (48.8)	127 (52.5)	
No	157 (48.5)	42 (51.2)	115 (47.5)	
Hypertension				0.121
Yes	184 (56.8)	53 (64.6)	131 (54.1)	
No	140 (43.2)	29 (35.4)	111 (45.9)	
Diabetes				0.172
Yes	105 (32.4)	32 (39)	73 (30.2)	
No	219 (67.6)	50 (61)	169 (69.8)	
Hyperlipemia				0.655
Yes	77 (23.8)	21 (25.6)	56 (23.1)	
No	247 (76.2)	61 (74.4)	186 (76.9)	
Anxiety				<0.001
Yes	231 (71.3)	36 (43.9)	57 (23.6)	
No	93 (28.7)	46 (56.1)	185 (76.4)	
Follow-up time	164 (144–180)	101.5 (62.5–135)	172 (158.5–180)	<0.001

Follow-up time in days. Group I (*n* = 82), Group II (*n* = 242). Spouse means spouse's health, well means healthy spouse, unwell means unhealthy spouse, none means no spouse.

Regarding surgical information, there were no differences between the two groups in terms of ischemic time before surgery and whether the myocardial infarction site was an anterior wall infarction (*P* > 0.05). Significant differences were found between the recurrent angina group and the non-recurrent group in terms of Killip classification of myocardial infarction, the number of coronary artery lesions, the number of stents implanted, intraoperative thrombolysis, postoperative revascularization status, TIMI score, and TMBG score (*P* < 0.05). See Table 2 for details.

**Table 2:** Comparison of surgical data between the two groups of patients.

Characteristics	Totality	Group I	Group II	*P*
Ischemic time				0.087
0–6	201 (62.0)	59 (72.0)	142 (58.7)	
6–12	56 (17.3)	12 (14.6)	44 (18.2)	
>12	67 (20.7)	11 (13.4)	56 (23.1)	
AWMI				0.203
Yes	150 (46.3)	43 (52.4)	107 (44.2)	
No	174 (53.7)	39 (47.6)	135 (55.8)	
Killip grading				<0.001
1	207 (63.9)	33 (40.2)	174 (71.9)	
2	72 (22.2)	17 (20.7)	55 (22.7)	
≥3	45 (13.9)	32 (39.1)	13 (5.4)	
Coronary lesions				<0.001
1	170 (52.5)	30 (36.6)	140 (57.9)	
2	120 (37.0)	32 (39.0)	88 (36.4)	
≥3	34 (10.5)	20 (24.4)	14 (5.8)	
Stents implantation				0.022
1	194 (59.9)	46 (56.1)	148 (61.2)	
2	93 (28.7)	24 (29.3)	69 (28.5)	
≥3	15 (4.6)	1 (1.2)	14 (5.8)	
0	22 (6.8)	11 (13.4)	11 (4.5)	
Thrombolysis				0.012
Yes	208 (64.2)	39 (47.6)	77 (31.8)	
No	116 (35.8)	43 (53.4)	165 (38.2)	
Revascularization mode				<0.001
SCLCR	170 (52.5)	30 (36.6)	140 (57.9)	
MCLCR	47 (14.5)	11 (13.4)	36 (14.9)	
MCLIR	107 (33.0)	41 (50.0)	66 (27.3)	
TIMI				<0.001
1	4 (1.2)	3 (3.7)	1 (0.4)	
2	72 (22.3)	29 (35.4)	43 (17.8)	
3	248 (76.5)	50 (61.0)	198 (81.8)	
TMBG				<0.001
1	4 (1.2)	3 (3.7)	1 (0.4)	
2	76 (23.5)	27 (32.9)	44 (18.2)	
3	244 (75.3)	52 (63.4)	197 (81.4)	

Ischemic time in hours. AWMI: anterior wall myocardial infarction. Coronary lesions: Number of coronary lesions. Stents implantation: Number of coronary stents implanted. Revascularization modalities include the following classifications: SCLCR means complete revascularization of a single vessel, MCLCR means complete revascularization of multiple vessels, MCLIR means incomplete revascularization of multiple vessels.

Concerning postoperative changes and medication, there were no noticeable differences between the two groups in adverse events during hospitalization and medication use (*P* > 0.05). However, there was a significant difference between the two groups in terms of chest pain relief within 36 h post-surgery. The group with recurrent chest pain had a higher proportion of patients who did not experience chest pain relief than the group without recurrence. The difference was statistically significant (*P* < 0.05), as illustrated in Table 3.

**Table 3:** Postoperative condition and medication in both groups.

Characteristics	Totality	Group I	Group II	*P*
Chest pain 36 h after surgery				<0.001
Remission	215 (66.4)	39 (47.6)	176 (72.7)	
Nonremission	109 (33.6)	43 (52.4)	66 (27.3)	
In-hospital adverse events				0.326
Yes	101 (31.2)	22 (26.8)	79 (32.6)	
No	223 (68.8)	60 (73.2)	163 (67.4)	
Postoperative medication				
Double anti-plate	324 (100)	82 (100)	242 (100)	—
Statins	324 (100)	82 (100)	242 (100)	—
ACEI/ARB	122 (37.7)	34 (41.5)	88 (36.4)	0.431
Beta blockers	120 (37.0)	28 (34.1)	92 (38.0)	0.597
Nitrates	155 (47.8)	37 (45.1)	118 (48.8)	0.610

In-hospital adverse events: Including severe hypotension, acute heart failure, cardiac arrhythmia, shock, and infection of the puncture incision. ACEI/ARB: Angiotensin-converting enzyme inhibitors and angiotensin II receptor antagonists.

### 4.2. COX regression analysis

Based on follow-up results, whether angina recurred was taken as the dependent variable, and significant baseline data, as well as some clinical data with differences, were included as independent variables in the COX regression model. The univariate COX regression model indicated that patient anxiety, spouse illness, Killip classification, the number of coronary lesions, stent implants, intraoperative thrombolysis, revascularization method, and TIMI score were all related to recurrent angina. After testing, some variables, including the number of coronary lesions, stent implants, postoperative revascularization status, TIMI score, and TMBG score, demonstrated strong interactivity. To eliminate multicollinearity, some items were excluded, retaining those with less correlation with other research variables. Multivariate COX regression analysis showed that patient anxiety and spouse illness were both risk factors for recurrent angina after discharge. The HR (95% CI) were 1.641 (1.050–2.566) and 2.624 (1.618–4.255), respectively. Refer to Table 4.

**Table 4:** COX regression.

Characteristics		Univariate		mnivariate	
		HR (95% CI)	*P*	HR (95% CI)	*P*
Sex	Female	Reference		Reference	
	Male	0.812 (0.494–1.333)	0.410	0.889 (0.526–1.503)	0.661
Age	<65	Reference		Reference	
	≥65	1.263 (0.816–1.954)	0.294	1.251 (0.801–1.953)	0.325
BMI	≤24	Reference		Reference	
	>24	1.081 (0.698–1.675)	0.727	0.885 (0.565–1.385)	0.592
Spouse	Well	Reference		Reference	
	Unwell	2.498 (1.582–3.945)	<0.001	2.624 (1.618–4.255)	<0.001
	None	2.247 (1.048–4.817)	0.037	1.676 (0.753–3.732)	0.206
Hypertension	No	Reference		—	
	Yes	1.580 (0.959–3.372)	0.075		
Diabetes	No	Reference		—	
	Yes	1.401 (0.899–2.184)	0.136		
Hyperlipemia	No	Reference		—	
	Yes	1.120 (0.682–1.839)	0.654		
Anxiety	No	Reference		Reference	
	Yes	2.106 (1.361–3.258)	<0.001	1.641 (1.050–2.566)	0.030
Ischemic time	0–6	Reference		–	
	6–12	0.689 (0.370–1.282)	0.240		
	>12	0.540 (0.284–1.027)	0.060		
Killip grading	1	Reference		Reference	
	2	1.571 (0.875–2.821)	0.130	1.612 (0.859–2.903)	0.112
	≥3	6.134 (3.761–10.004)	<0.001	4.983 (2.965–8.377)	<0.001
Coronary lesions	1	Reference		–	
	2	1.537 (0.934–2.529)	0.091		
	≥3	4.356 (2.468–7.689)	<0.001		
Stents implantation	1	Reference		Reference	
	2	1.099 (0.671–1.800)	0.709	1.055 (0.635–1.755)	0.835
	≥3	2.587 (1.339–4.998)	0.005	1.432 (0.036–1.943)	0.192
	0	0.256 (0.035–1.858)	0.178	0.266 (0.682–1.839)	0.308
Thrombolysis	No	Reference		Reference	
	Yes	1.739 (1.128–2.683)	0.012	1.429 (0.906–2.254)	0.125
Revascularization mode	MCLCR	Reference		–	
	SCLCR	1.737 (0.893–3.380)	0.104		
	MCLIR	0.735 (0.368–1.467)	0.383		
TIMI	3	Reference		–	
	2	7.122 (2.216–22.889)	<0.001		
	1	2.170 (1.373–3.430)	<0.001		

Ischemic time in hours. Coronary lesions: Number of coronary lesions. Stents implantation: Number of coronary stents implanted. Revascularization modalities include the following classifications: SCLCR means complete revascularization of a single vessel, MCLCR means complete revascularization of multiple vessels, MCLIR means incomplete revascularization of multiple vessels. Spouse means spouse's health, well means healthy spouse, unwell means unhealthy spouse, none means no spouse.

### 4.3. Propensity score matching (PSM) results

To eliminate the impact of endogeneity bias and explore the relationship between anxiety and post-discharge recurrent angina, this study used PSM to perform a 1:1 match between the two groups of patients. Matching was done between the anxiety and non-anxiety groups, primarily based on major baseline data and some clinical data with differences. Table 5 provides patient information before and after matching. As shown in Table 5, before matching, there were a total of 324 patients, with 93 having anxiety disorders and 231 without. After matching, the total number of patients was 162, with 81 each in the anxiety and non-anxiety groups. After matching, there were no significant differences in major baseline data and some pre-matching clinical data with differences between the two groups (*P* > 0.05).

**Table 5:** Propensity score matching (PSM).

Characteristics		Before PSM			After PSM		
		Anxiety	Anxiety free	*P*	Anxiety	Anxiety free	*P*
Sex	Male	69 (74.2)	183 (79.2)	0.376	62 (76.5)	64 (79.0)	0.850
	Female	24 (25.8)	48 (20.8)		19 (23.5)	17 (21.0)	
Age	≥65	51 (54.8)	113 (48.9)	0.390	44 (54.3)	40 (49.4)	0.637
	<65	42 (45.2)	118 (51.1)		37 (45.7)	41 (50.6)	
BMI	>24	50 (53.8.)	129 (55.8)	0.805	46 (56.8)	48 (59.3)	0.874
	≤24	43 (46.2)	102 (44.2)		35 (43.2)	33 (40.7)	
Spouse	Well	57 (61.3)	151 (65.4)	0.292	51 (63.0)	52 (64.2)	0.318
	Unwell	32 (34.4)	82 (26.8)		28 (34.6)	23 (28.4)	
	No	4 (4.3)	18 (7.8)		2 (2.5)	6 (7.4)	
Hypertension	Yes	53 (57.0)	131 (56.7)	0.963	47 (58.0)	49 (60.5)	0.873
	No	40 (43.0)	100 (43.3)		34 (42.0)	32 (39.5)	
Diabetes	Yes	34 (36.6)	71 (30.7)	0.359	28 (34.6)	26 (32.1)	0.868
	No	59 (63.4)	160 (69.3)		53 (65.4)	55 (67.9)	
Hyperlipemia	Yes	20 (78.5)	57 (24.7)	0.568	20 (24.7)	18 (22.2)	0.427
	No	73 (21.5)	174 (75.3)		61 (75.3)	63 (77.8)	
Ischemic time	0–6	60 (64.5)	141 (61.0)	0.415	51 (63.0)	55 (67.9)	0.823
	6–12	18 (19.4)	38 (16.5)		16 (19.8)	14 (17.3)	
	>12	15 (16.1)	52 (22.5)		14 (17.2)	12 (14.8)	
Killip grading	1	56 (60.2)	151 (65.4)	0.104	55 (67.9)	43 (53.1)	0.142
	2	18 (19.4)	54 (23.4)		14 (17.3)	25 (28.4)	
	≥3	19 (20.4)	26 (11.3)		12 (14.8)	15 (18.5)	
Coronary lesions	1	40 (43.0)	130 (56.3)	0.035	40 (49.4)	38 (46.9)	0.595
	2	38 (40.9)	82 (35.5)		31 (38.3)	28 (34.6)	
	≥3	15 (16.1)	19 (8.2)		10 (12.3)	15 (18.5)	
Stents implantation	1	57 (61.3)	137 (59.3)	0.491	53 (65.4)	49 (60.5)	0.503
	2	23 (24.7)	70 (30.3)		18 (22.2)	25 (30.9)	
	≥3	9 (9.7)	13 (5.6)		7 (8.6)	6 (7.4)	
	0	4 (4.3)	11 (4.8)		3 (3.7)	1 (1.2)	
Thrombolysis	Yes	55 (59.1)	153 (66.2)	0.250	32 (39.5)	32 (39.5)	1.000
	No	38 (40.9)	78 (33.8)		49 (60.5)	49 (60.5)	
Revascularization mode	SCLCR	40 (43.0)	130 (56.3)	0.087	40 (49.4)	38 (46.9)	0.905
	MCLCR	15 (40.9)	32 (13.8)		11 (13.6)	10 (12.3)	
	MCLIR	38 (16.1)	69 (29.9)		30 (37.0)	33 (40.8)	
TIMI	1	2 (2.2)	2 (0.9)	<0.001	0 (0)	1 (1.2)	0.596
	2	34 (36.6)	38 (16.5)		24 (29.6)	20 (24.7)	
	3	57 (61.3)	191 (82.7)		57 (70.4)	60 (74.1)	

Ischemic time in hours. Coronary lesions: Number of coronary lesions. Stents implantation: Number of coronary stents implanted. Revascularization modalities include the following classifications: SCLCR means complete revascularization of a single vessel, MCLCR means complete revascularization of multiple vessels, MCLIR means incomplete revascularization of multiple vessels. Spouse means spouse's health, well means healthy spouse, unwell means unhealthy spouse, none means no spouse.

### 4.4. COX regression analysis after PSM matching

Multivariable regression analysis post-propensity score matching showed that spouse illness, anxiety, and Killip ≥3 were all related to recurrent angina. The effect of anxiety was particularly significant (HR = 2.094, 95% CI = 1.248–3.514, *P* = 0.005), consistent with the pre-matching results. See Table 6 for details.

**Table 6:** Multifactor COX regression after PSM.

Characteristics		*N*	HR (95% CI)	*P*
Sex	Female	36	Reference	
	Male	126	0.786 (0.421–1.465)	0.448
Age	<65	78	Reference	
	≥65	84	1.245 (0.753–2.059)	0.393
BMI	≤24	68	Reference	
	>24	94	1.307 (0.771–2.218)	0.32
Spouse	Well	103	Reference	
	Unwell	51	3.148 (1.846–5.367)	<0.001
	None	8	3.042 (1.120–8.261)	0.029
Anxiety	No	81	Reference	
	Yes	81	2.094 (1.248–3.514)	0.005
Killp rating	1	98	Reference	
	2	37	0.995 (0.513–1.932)	0.989
	≥3	27	2.981 (1.681–5.284)	<0.001
Coronary lesions	1	78	Reference	
	2	59	0.792 (0.446–1.407)	0.427
	≥3	25	0.999 (0.502–1.985)	0.997
Thrombolysis	No	98	Reference	
	Yes	64	1.613 (0.976–2.667)	0.062
TIMI	3	117	Reference	
	2	45	1.193 (0.703–2.025)	0.514

Spouse means spouse's health, well means healthy spouse, unwell means unhealthy spouse, none means no spouse. Coronary lesions: Number of coronary lesions. Stents implantation: Number of coronary stents implanted.

### 4.5. Subgroup analysis

**Figure 2:**
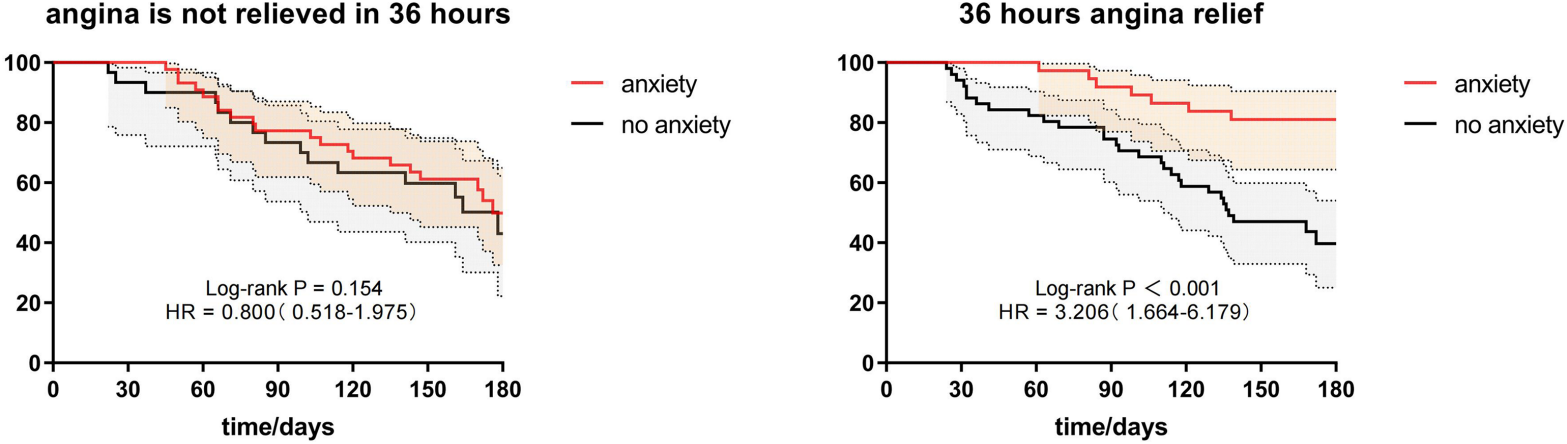
Subgroup analysis of whether chest pain was relieved at 36 h postoperatively.

Comparing baseline data between the two groups, we found a statistically significant difference in postoperative 36-hour chest pain relief among discharged patients (see Table 3). To evaluate whether the lack of short-term postoperative chest pain relief and recurrent chest pain after discharge were related to patient anxiety, we divided the patients matched by PSM into two groups based on whether they experienced chest pain relief within 36 h post-surgery: the chest pain relief group (*n* = 88) and the no chest pain relief group (*n* = 74). Subgroup analysis results indicated that anxiety was associated with post-discharge recurrent angina at both the 36-hour post-surgery chest pain relief (HR = 2.810, 95% CI = 1.205–6.552, *P* = 0.017) and the 36-hour post-surgery no chest pain relief (HR = 1.988, 95% CI = 1.074–3.679, *P* = 0.029) levels. Details are in Table 7 and Figure 2.

**Table 7:** Subgroup analysis of whether chest pain was relieved at 36 h postoperatively.

Characteristics	Anxiety	*N*	HR (95% CI)	*P*
36 h angina relief	No	51	Reference	
	Yes	37	2.810 (1.205–6.552)	0.017
Angina is not relieved in 36 h	No	30	Reference	
	Yes	44	1.988 (1.074–3.679)	0.029
Totality	No	81	Reference	
	Yes	81	2.232 (1.344–3.706)	0.002

**Figure 3:**
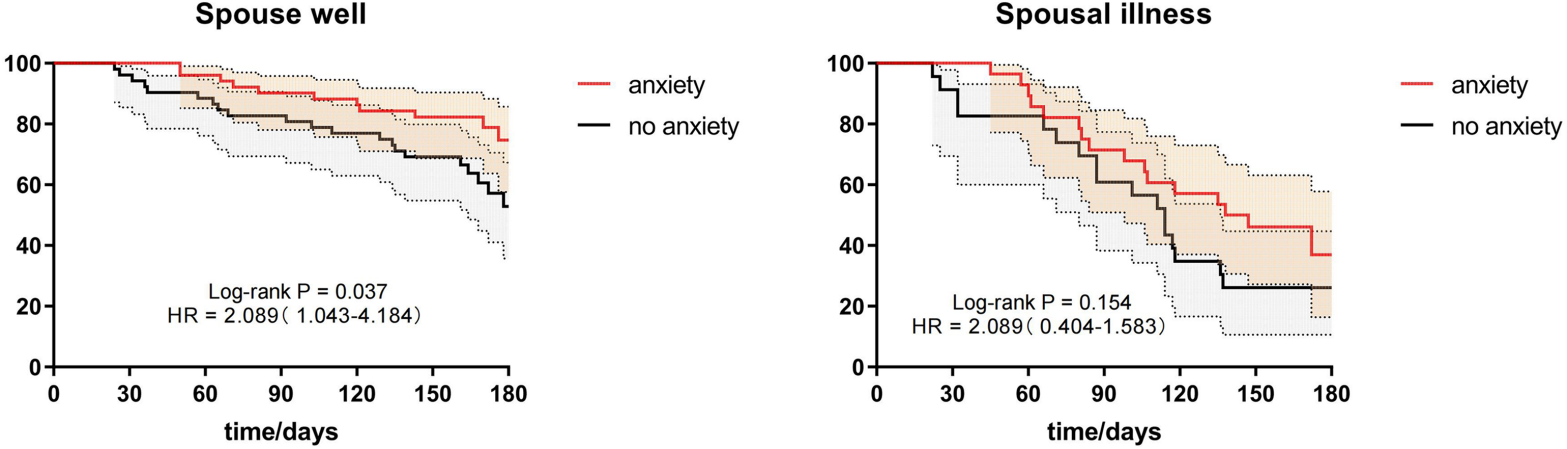
Subgroup analysis of spousal health status.

To explore whether differences in the health status of a patient's spouse might influence recurrent angina and if it is related to patient anxiety, we divided the patients based on their spouse's health status into three groups: spouse healthy, spouse ill, and no spouse. Due to sample size limitations, the no-spouse group, which had a smaller number of patients after PSM matching, was excluded from the discussion. Subgroup analysis suggested that for patients in the spouse healthy group, anxiety might be a factor for recurrent angina within 6 months post-discharge (HR = 2.129, 95% CI = 1.26–4.418, *P* = 0.042). The recurrence of angina in patients in the spouse ill group was not related to patient anxiety (*P* > 0.05). Details are in Table 8 and Figure 3.

**Table 8:** Subgroup analysis of spousal health status.

Characteristics	Anxiety	*N*	HR (95% CI)	*P*
Spouse well	No	52	Reference	
	Yes	51	2.129 (1.26–4.418)	0.042
Spousal illness	No	23	Reference	
	Yes	28	1.638 (0.822–3.264)	0.160
Mateless	No	6	Reference	
	Yes	2	——	0.249
Totality	No	81	Reference	
	Yes	81	2.055 (1.265–3.341)	0.004

**Figure 4:**
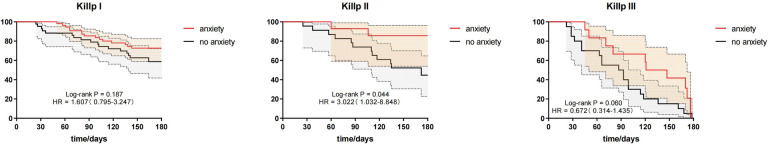
Subgroups analysis of different killip grading subgroups.

To study whether the severity of a patient's condition leading to recurrent angina post-discharge was related to anxiety, subgroup analysis was conducted based on the Killip classification to examine whether recurrent angina in patients with different Killip classifications was associated with anxiety. The results showed that, at different Killip classification levels, the recurrence of angina within 6 months post-STEMI PCI was not related to whether the patient was anxious. Refer to Table 9 and Figure 4.

**Table 9:** Subgroups analysis of different killip grading subgroups.

Characteristics	Anxiety	*N*	HR (95% CI)	*P*
Killp I	No	43	Reference	
	Yes	55	1.590 (0.794–3.184)	0.191
Killp II	No	23	Reference	
	Yes	14	4.155 (0.926–18.642)	0.063
Killp III	No	15	Reference	
	Yes	12	2.087 (0.949–4.589)	0.067
Totality	No	81	Reference	
	Yes	81	1.912 (1.172–3.118)	0.009

## 5. Discussion

Cardiovascular diseases have consistently been the leading cause of human mortality, with Acute Myocardial Infarction (AMI) being among the most severe types. ST-segment elevation myocardial infarction (STEMI) is the most common type of AMI. The fastest and most effective clinical treatment for STEMI is Percutaneous Coronary Intervention (PCI). However, post-PCI angina recurrence poses a challenge to both STEMI patients and clinicians due to its complex etiology and the sheer number of affected individuals. Despite advancements in medical treatment, many patients can't effectively control its recurrence even with regular medication (15). In this study, we primarily investigated the risk factors of angina recurrence after PCI in STEMI patients and explored the impact of anxiety on post-discharge angina recurrence. Since all participants in this study had undergone PCI following STEMI, no significant differences in baseline data were identified. Factors influencing angina recurrence after discharge mainly included anxiety levels, spouse's health status, and differences in surgical data. To better understand the impact of anxiety on angina recurrence, we treated patient anxiety as an independent variable and angina recurrence as the dependent variable, applying Propensity Score Matching (PSM) to match baseline data and significantly different surgical data to reduce bias. Multivariate COX regression analysis before matching showed that patient anxiety status, spouse's illness, and a Killip classification of ≥3 were factors for post-PCI angina recurrence. Post-matching COX regression results supported these findings. Before including surgical data as control variables in the COX regression model, we conducted an interaction test on some surgical data. Strong multicollinearity was found among variables like TIMI grade, vascular reconstruction methods, the number of coronary lesions, and the number of stents implanted. To ensure the accuracy of the regression model results, we excluded some variables, retaining those with minimal interaction with other variables. During the research, we found that pain relief within 36 h post-surgery was significantly different at the time of discharge. To determine if this was related to the patient's anxiety status, we grouped by pain relief within 36 h post-PCI and carried out a subgroup analysis. The results showed that anxiety was related to post-discharge angina recurrence regardless of pain relief status within 36 h after surgery. Subsequent subgroup analyses based on the COX regression results suggest that among patients with healthy spouses, post-discharge angina recurrence was related to the patient's anxiety status. Contrary to subjective feelings, angina occurrence after discharge in patients with sick spouses and those with various Killip classifications was not related to anxiety. This result might be due to insufficient sample size, but combined with COX regression results, it still reflects that the sickness status of the patient's spouse and a higher Killip classification are independent risk factors for post-discharge angina recurrence after PCI.

The negative impact of anxiety disorders on the cardiovascular system has already been discussed in our literature review. Besides increasing the activity of the sympathetic nervous system, leading to faster heart rates, inducing arrhythmias, elevating blood pressure, causing damage to blood vessel walls, and accelerating the process of arteriosclerosis, anxiety is also linked to poor health behaviors such as smoking, excessive drinking, reduced physical activity, and unhealthy diets. All these increase the risk of cardiovascular diseases (16). The theoretical basis of this study is grounded in the belief that anxiety has detrimental effects on the cardiovascular system. However, existing theories also suggest that having a cardiovascular disease can induce anxiety in patients (17, 18). Given that AMI is one of the most severe cardiovascular diseases, patients inevitably develop fears about mortality and further heart attacks post-diagnosis. Considering the reduced physical capabilities post-surgery and the long-term medications, the financial burden on the family increases. Preventing another heart attack might also require changes to existing lifestyles, possibly limiting social interactions. All these factors could induce anxiety. It's evident that anxiety and cardiovascular diseases have a mutually reinforcing relationship. This mutual impact is unfavorable for clinicians and needs mitigation. By deeply exploring this issue, we hope to see more similar studies providing more precise risk assessment and management strategies, thereby further optimizing the treatment and rehabilitation of STEMI patients.

There are some limitations to our study. Firstly, the insufficient sample size can lead to inadequate test power, increasing the risk of Type II errors. Secondly, gauging emotional status changes post-discharge is challenging. The risk of anxiety disorders may increase after a STEMI episode, and some patients may develop new anxiety symptoms post-discharge, which might be overlooked during the in-hospital Hamilton Anxiety Rating Scale assessment. To address these challenges, more prospective studies with larger sample sizes are needed.

## Data Availability

The original contributions presented in the study are included in the article/Supplementary Material, further inquiries can be directed to the corresponding author.

## References

[1] llrich H Olschewski M Münzel T Gori T. Coronary in-stent restenosis: predictors and treatment. Dtsch Arztebl Int. (2021) 118(38):637–44. 10.3238/arztebl.m2021.0254 34379053 PMC8715314

[2] Radley C Berry C. Definition and epidemiology of coronary microvascular disease. J Nucl Cardiol. (2022) 29(4):1763–75. 10.1007/s12350-022-02974-x 35534718 PMC9345825

[3] l-Lamee RK Nowbar AN Francis DP. Percutaneous coronary intervention for stable coronary artery disease. Heart. (2019) 105(1):11–9. 10.1136/heartjnl-2017-312755 30242142

[4] De Luca L Rosano GMC Spoletini I. Post-percutaneous coronary intervention angina: from physiopathological mechanisms to individualized treatment. Cardiol J. (2022) 29(5):850–7. 10.5603/CJ.a2021.0042 33843042 PMC9550331

[5] En-Yehuda O Kazi DS Bonafede M Wade SW Machacz SF Stephens LA Angina and associated healthcare costs following percutaneous coronary intervention: a real-world analysis from a multi-payer database. Catheter Cardiovasc Interv. (2016) 88(7):1017–24. 10.1002/ccd.26365 26774951

[6] Iwaszczuk P Łosiak W Szczeklik W Musiałek P. Patient periprocedural stress in cardiovascular medicine: friend or foe? Adv Interv Cardiol. (2021) 17(3):259–71. 10.5114/aic.2021.109176 PMC8596718 34819962

[7] Alkstedt D Lundberg I Hemmingsson T. Childhood socio-economic position and risk of coronary heart disease in middle age: a study of 49,321 male conscripts. Eur J Public Health. (2011) 21(6):713–8. 10.1093/eurpub/ckq158 21051471

[8] I B Ding X. Observation and analysis of 126 cases of coronary heart disease unstable angina pectoris complicated with anxiety. Medical Food Therapy and Health. (2020) 18(20):62–5.

[9] Brouwers RW Kraal JJ Traa SC. Effects of cardiac telerehabilitation in patients with coronary artery disease using a personalised patient-centred web application: protocol for the SmartCare-CAD randomised controlled trial. BMC Cardiovasc Disord. (2017) 17(1):46. 10.1186/s12872-017-0477-6 28143388 PMC5282829

[10] Zhang S Zhong Y Wang L. STEP Study Group. Anxiety, home blood pressure monitoring, and cardiovascularevents among older hypertension patients during the COVID-19 pandemic. Hypertens Res. (2022) 45(5):856–65. 10.1038/s41440-022-00852-0 35064249 PMC8778505

[11] Chialà O Vellone E Klompstra L Jaarsma T. Relationships between exercise capacity and anxiety, depression, and cognition in patients with heart failure. Heart Lung. (2018) 47(5):465–70. 10.1016/j.hrtlng.2018.07.010 30087002

[12] Torabizadeh C Rousta S Parvizi MM. Efficacy of education delivery through multimedia and text messaging on the psychological parameters of patients scheduled for coronary angiography: a single-blind randomized controlled clinical trial. BMC Cardiovasc Disord. (2021) 21(1):3. 10.1186/s12872-020-01820-7 33397300 PMC7784265

[13] Thomas M Jones PG Arnold SV Spertus JA. Interpretation of the Seattle angina questionnaire as an outcome measure in clinical trials and clinical care: a review. JAMA Cardiol. (2021) 6(5):593–9. 10.1001/jamacardio.2020.7478 33566062 PMC8651216

[14] Zhu C Zhang Y Wang T Zhu DM. Vitamin D supplementation improves anxiety but not depression symptoms in patients with vitamin D deficiency. Brain Behav. (2020) 10(11):e01760. 10.1002/brb3.1760 32945627 PMC7667301

[15] Peterson BE Bhatt DL Steg PG Ballantyne CM. REDUCE-IT Investigators. Treatment with icosapent ethyl to reduce ischemic events in patients with prior percutaneous coronary intervention: insights from REDUCE-IT PCI. J Am Heart Assoc. (2022) 11(6):e022937. 10.1161/JAHA.121.022937 35261279 PMC9075300

[16] Broers ER Gavidia G Wetzels M Ribas V Ayoola I Piera-Jimenez J Usefulness of a lifestyle intervention in patients with cardiovascular disease. Am J Cardiol. (2020) 125(3):370–5. 10.1016/j.amjcard.2019.10.041 31761149

[17] Wen Y Yang Y Shen J Luo S. Anxiety and prognosis of patients with myocardial infarction: a meta-analysis. Clin Cardiol. (2021) 44(6):761–70. 10.1002/clc.23605 33960435 PMC8207975

[18] Zhou S J Zhang J. Research progress on the relationship between anxiety and depression and its evaluation and cardiovascular diseases. Journal of Cardiovascular Rehabilitation Medicine. (2021) 30(3):335–40. 10.3969/j.issn.1008-0074.2021.03.22

